# Broad ligament pregnancy case, the ectopic gestational sac was very close to the ureter

**DOI:** 10.1002/ccr3.6236

**Published:** 2022-08-09

**Authors:** Jie Gao, Yu Wang

**Affiliations:** ^1^ Department of Gynecology Bengbu Third People's Hospital BengBu city China

**Keywords:** broad ligament, ectopic pregnancy, laparoscopy, ureter

## Abstract

A broad ligament pregnancy had been managed by laparoscopy. A 2‐cm ectopic pregnancy sac was located in the posterior lobe of the left broad ligament. We identified and separated the ipsilateral ureter during the operation because the ectopic gestational sac was very close to the ureter.

## Graphical abstract

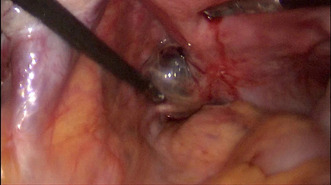

The patient was hospitalized on July 20, 2018, with menolipsis for 61 days and no other symptoms or physical signs. An abdominal pregnancy was diagnosed by pelvic ultrasound (Figures 1, 2, 3). We performed laparoscopic surgery on promptly. A 2 cm ectopic pregnancy sac was located in the posterior lobe of the left broad ligament (still image). We identified and separated the ipsilateral ureter during the operation because the ectopic gestational sac was very close to the ureter (Video S1).^1^, ^2^ She recovered quickly after the surgery, and there were no problems during the 6 month follow‐up.

**FIGURE 1:**
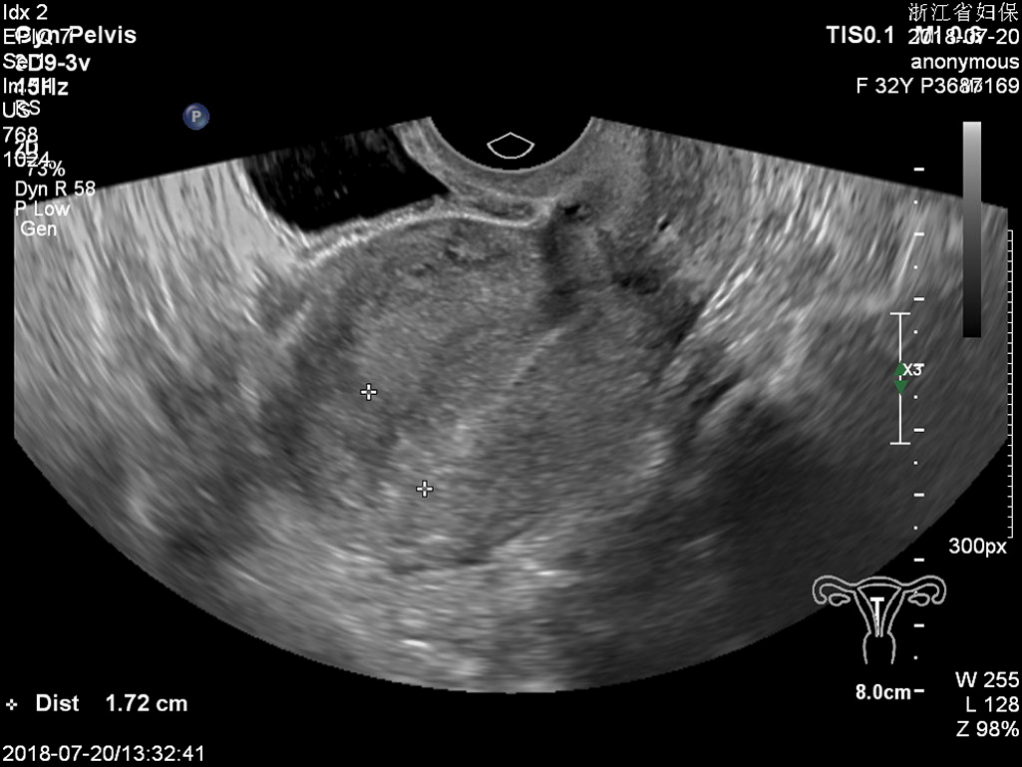
Thickened endometrium is seen with no pregnancy sac in the uterine cavity

**FIGURE 2:**
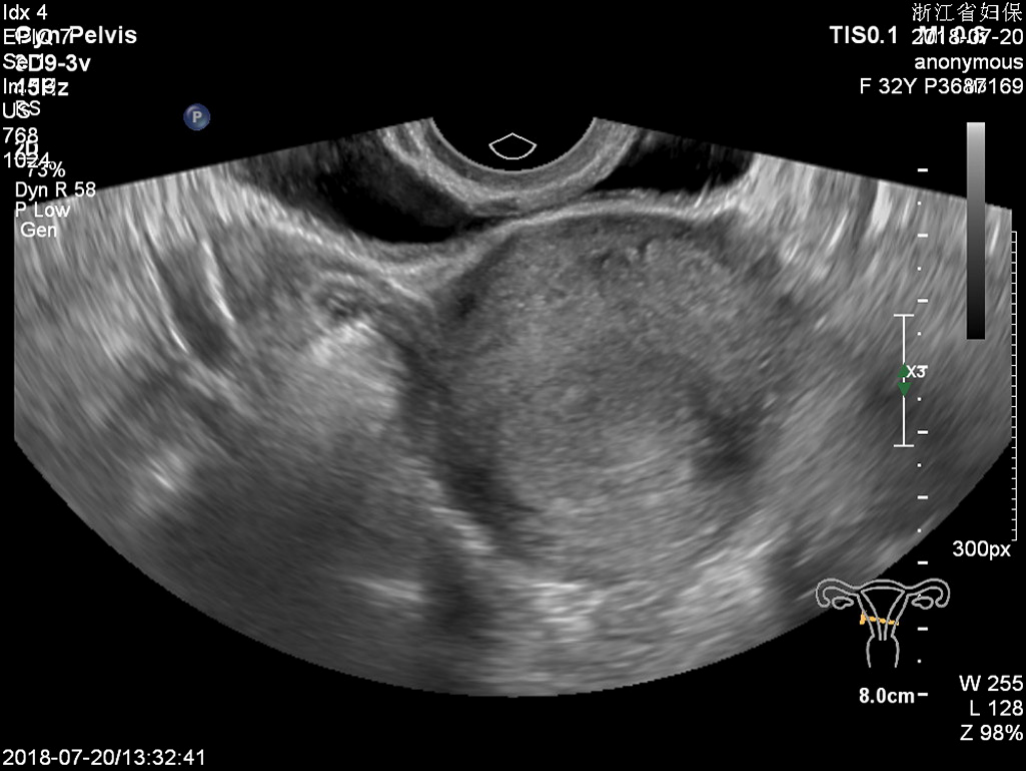
A 3.5 × 2.3 × 2.0 cm uneven echoic mass is seen at the left side of the uterine isthmus

**FIGURE 3:**
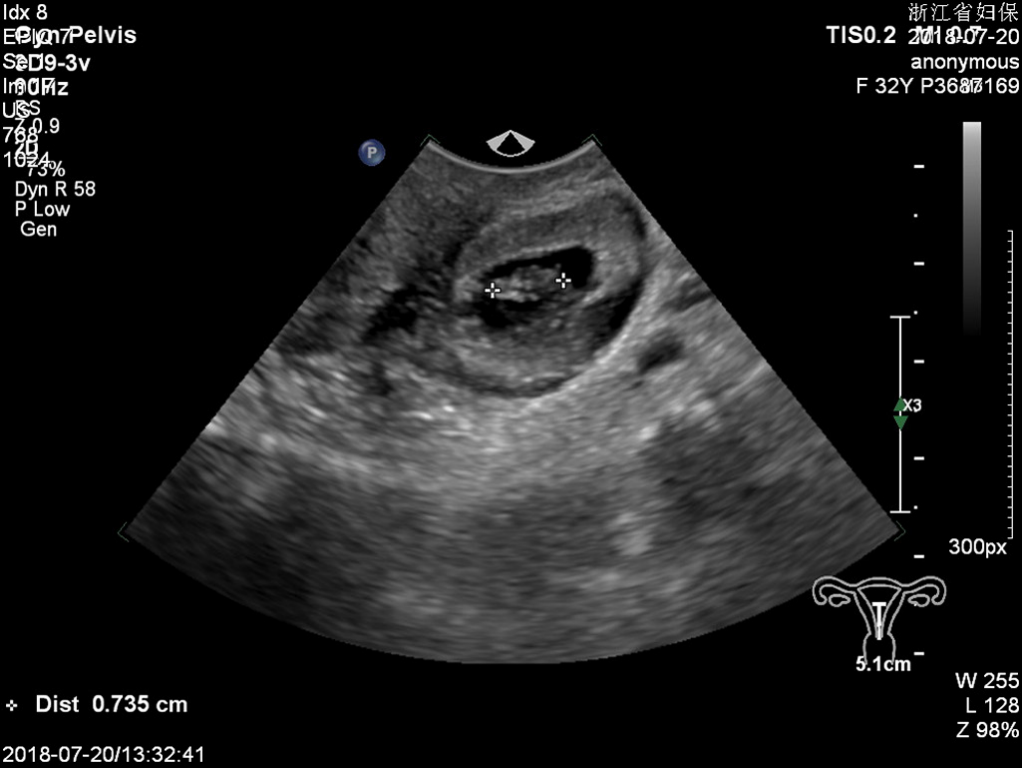
Dark area, about 2.2 cm long, is seen in the parametrial uneven echoic mass. A yolk cyst‐like echo is seen in the dark area without a heartbeat

## AUTHOR CONTRIBUTIONS

Jie Gao did substantial contributions to the conception or design of the work, drafting the work, or revising it critically for important intellectual content. Yu Wang made final approval of the version to be published, agreement to be accountable for all aspects of the work in ensuring that questions related to the accuracy or integrity of any part of the work are appropriately investigated and resolved.

## CONFLICT OF INTEREST

The authors declare no conflicts of interest.

## ETHICAL APPROVAL

The patient had provided written informed consent for publication of their data. Institutional Review Board approval was not required.

## STATEMENT

This manuscript has not been published or presented elsewhere and is not under consideration by another journal.

## CONSENT

The patient had provided written informed consent for publication of their data. Institutional Review Board approval was not required.

## Supporting information


Video S1

## Data Availability

Data sharing not applicable to this article as no datasets were generated or analysed during the current study.
